# Weil’s Disease—Immunopathogenesis, Multiple Organ Failure, and Potential Role of Gut Microbiota

**DOI:** 10.3390/biom12121830

**Published:** 2022-12-07

**Authors:** Pavlo Petakh, Vitaliia Isevych, Aleksandr Kamyshnyi, Valentyn Oksenych

**Affiliations:** 1 Department of Microbiology, Virology, and Immunology, I. Horbachevsky Ternopil National Medical University, 46001 Ternopil, Ukraine; 2 Department of Biochemistry and Pharmacology, Uzhhorod National University, 88000 Uzhhorod, Ukraine; 3 Institute of Clinical Medicine, University of Oslo, 0318 Oslo, Norway

**Keywords:** leptospirosis, dysbiosis, Weil’s disease

## Abstract

Leptospirosis is an important zoonotic disease, causing about 60,000 deaths annually. In this review, we have described in detail the immunopathogenesis of leptospirosis, the influence of cytokines, genetic susceptibility on the course of the disease, and the evasion of the immune response. These data are combined with information about immunological and pathomorphological changes in the kidneys, liver, and lungs, which are most affected by Weil’s disease. The review also suggests a possible role of the gut microbiota in the clinical course of leptospirosis, the main mechanisms of the influence of gut dysbiosis on damage in the liver, kidneys, and lungs through several axes, i.e., gut-liver, gut-kidney, and gut-lungs. Modulation of gut microbiota by probiotics and/or fecal microbiota transplantation in leptospirosis may become an important area of scientific research.

## 1. Introduction

Leptospirosis is a re-emerging zoonosis, caused by *Leptospira* spp. It is estimated to infect more than a million people with approximately 60,000 deaths annually [1]. Disease is usually transmitted by contact with the urine of a host carrying the pathogen in water or soil, causing infection in humans through the skin or the gastrointestinal tract (GI) [2]. Human leptospirosis has a wide range of clinical manifestations, including mild, asymptomatic infections, as well as serious and life-threatening complications with multi-organ dysfunction, e.g., when kidneys, lungs, and liver are seriously damaged [3,4]. Weil’s syndrome (10% of cases), is a severe form of leptospirosis with a high mortality rate; it is characterized by hepatic dysfunctions associated with renal failure and hemorrhages [5]. Severe leptospirosis patients should receive early recognition and intensive medical care. The pathology of leptospirosis and the factors that cause severe leptospirosis are currently unclear [6,7] and antibiotic therapy is still the preferred treatment. However, due to the difficulty of early diagnosis of leptospirosis, some patients infected with *L. interrogans* often develop multiorgan dysfunction [8].

Both host factors and pathogens may play an important role in the pathogenesis of leptospirosis [9]. Induction of an inflammatory response by a pathogen can initiate destructive immune mechanisms leading to host tissue damage, sepsis, and death [10]. The extensive release of cytokines, including interleukin 6 (IL-6), interleukin 1 beta (IL-1β), and tumor necrosis factor-alpha (TNF-α), is known as a cytokine storm. Several studies have shown how cytokines contribute to pathogenesis and clinical manifestations of leptospirosis [11,12].

Gut dysbiosis is one of the potential factors that can affect the clinical course of leptospirosis [13]. Gut microbiota interacts with the host immune system in ways that influence the development of different diseases [14]. The gut microbiota generates a wide range of metabolites, such as short-chain fatty acids (SCFAs) or secondary bile acids [15]. SCFA has anti-inflammatory effects by inducing apoptosis and preserving the mucosal barriers to endotoxin infiltration [16]. There is growing evidence that there is a critical link between the gut microbiome and other organs most affected by leptospirosis, such as the liver, lungs, and kidneys [17,18]. This review aims to summarize and discuss some aspects of the immune response in leptospirosis, the pathogenesis of tissue damage, and relate these changes to possible gut dysbiosis.

## 2. Leptospira Immunity

**Figure 1:**
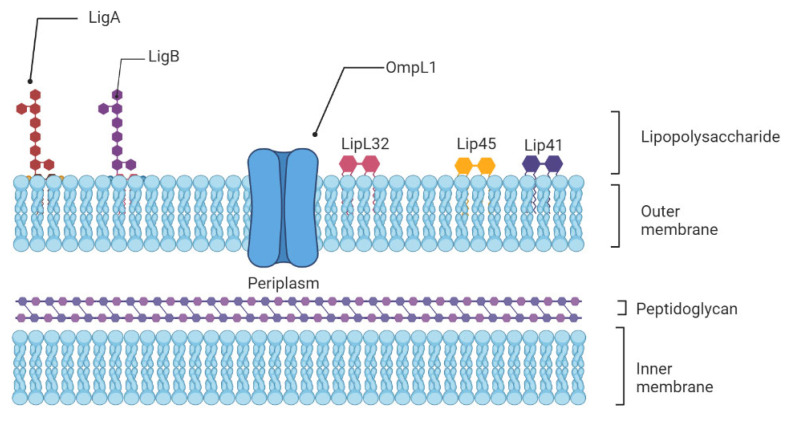
Schematic representation of the architecture of the leptospiral membrane. The inner membrane is closely associated with the peptidoglycan cell wall, which is overlaid by the outer membrane. Surface-exposed lipoproteins (LipL32, LigA, LigB), the transmembrane outer membrane protein porin L1 (OmpL1), and lipopolysaccharide are among the main components of the outer membrane.

The innate immune system is the first line of host defense and is essential in the early recognition and elimination of leptospires [9]. Microbial Pathogen-Associated Molecular Patterns (PAMPs) will be recognized by the Pattern Recognition Receptors (PRRs) expressed at the surface of innate immune cells, such as macrophages and dendritic cells (DCs), mainly the Toll-like receptors (TLRs) and nucleotide-binding oligomerization domain (NOD)-like receptors (NLRs) [19]. Among TLRs, TLR2 and TLR4 are the most studied in leptospirosis now. TLR4 can be activated by Lipopolysaccharides (LPS) from Gram-negative bacteria, which causes a pro-inflammatory cytokine- and chemokine-dependent response [20]. Leptospiral LPS is less endotoxic than Gram-negative LPS and activates human macrophages via TLR2 rather than TLR4 [21] (Figure 1).

This different recognition is attributed to the unusual composition of the leptospiral Lipid A moiety and could be a strategy that pathogenic Leptospira may use to avoid activation of immune cells, contributing to the initiation of the disease in humans [22]. In mice, which are resistant to leptospirosis, the LPS is recognized by both TLR2 and TLR4. Indeed, both TLR4 and TLR2 stimulation is important in controlling leptospirosis in mice. Infected with *L. interrogans*, double TLR2/TLR4 knockout mice died quickly from hepatic and renal failure [23]. Other receptors may mediate the binding of pathogens to host cells before their ingestion by phagocytic cells. DCs express diverse PRRs, including C-type lectins as DC-specific intercellular adhesion molecule-3-Grabbing Non-integrin (DC-SIGN), an endocytic receptor that recognizes high-mannose glycans and fucose-containing antigens on pathogens’ surfaces [24]. As polymorphonuclear neutrophils (PMNs) constitute the largest population of intravascular phagocytes, they are expected to play a critical role in leptospiral clearance. Neutrophils are major phagocytic cells that utilize a combination of reactive oxygen species (ROS), cytotoxic granules, antimicrobial peptides, and Neutrophil Extracellular Traps (NETs) to kill and degrade the invading pathogen [25].

The PAMPs/PRR association triggers an inflammatory cascade by activating multiple intracellular signaling pathways, including the NF-κB and activator protein 1 (AP-1) transcription factors, which in turn regulates the expression of cytokines, prostaglandins (PGs), and nitric oxide (NO) [26,27]. PGs and NO are pro-inflammatory molecules that enhance arterial dilatation and vascular permeability, both of which are essential for immune cell influx [28,29]. Pro-inflammatory cytokines include interleukins (IL)-1β, IL-6, IL-12, interferons (IFNs), and tumor necrosis factors (TNFs), as well as chemokines, which act as chemoattractants to recruit leucocytes to the site of tissue damage and infection [30]. In addition, activation of the complement system alternative route, particularly during the early hours of infection, is an essential aspect of the innate immune system and elimination of leptospires [31]. For example, saprophytic *L. biflexa* is killed within a few minutes in the presence of normal human serum in vitro, but it is not true for pathogenic Leptospira species which can survive, and are more resistant to the action of the complement system, especially if they are virulent [32]. Considering that immature DCs express TLR2 and that leptospires are able to interact with this receptor in human macrophages, it is particularly relevant to investigate the cross-talk between DC-SIGN and TLR pathways in DCs [33]. Therefore, further investigations on DCs activation and antigen presentation to T cells are required to elucidate the establishment of adaptive immune responses in leptospirosis.

## 3. Cytokines, Chemokines, Genetics, and Their Role in Severe Leptospirosis

Inflammatory cytokines and cytokine regulators participate in pathogen clearance without excessive inflammation-induced organ damage. Severe infectious diseases are often associated with a prolonged increase in pro-inflammatory IL-1β, TNF-α, IL-6 expression, or “cytokine storm”, causing persistent inflammation and followed by a massive and systemic production of anti-inflammatory cytokines [34]. Interestingly, the clinical hallmarks of severe leptospirosis can resemble septic shock, with multi-organ failure, hypotension, and death, which suggest that the development of severe leptospirosis could be associated with dysregulated inflammation [35]. There have been data obtained during clinical studies that confirm this hypothesis. The very first research of cytokines in human leptospirosis showed a significant increase in TNF-α levels from patient sera [36,37]. However, as TNF-α is an early phase cytokine, its levels are reduced or undetectable with disease progression [9]. Further investigations reported higher production of cytokines in severe compared to mild disease [10,11]. Serum levels of pro-inflammatory IL-6, chemokine IL-8, and anti-inflammatory IL-10 were significantly higher among patients that developed SPHS compared to non-SPHS leptospirosis patients [10]. In addition, concentrations of these cytokines were elevated in sera from patients with organ dysfunction compared to mild cases without organ involvement [38]. Additionally, in human leptospirosis, contradictory findings showed either greater IL-10 levels or no significant change in IL-10 levels [10,39]. Inflammatory response is restricted by regulatory cytokines, such as IL-4 or IL-13, promoting T helper (Th) lymphocyte differentiation toward Th2, suppressing tissue-damaging effects of sustained inflammation [40]. Serum levels of IL-4 were increased in human patients and frequencies in IL-4 and IL-4R receptor gene polymorphisms were significantly higher in leptospirosis patients compared to healthy subjects [41].

High levels of chemokines are found in susceptible hamsters, and are associated with organ damage and poor outcome [10,42]. Notably, patients with severe clinical signs had higher levels of CXCL8/IL-8 expression, and these patients had a higher mortality rate [39]. In addition, high concentrations of adhesion molecules (ICAM-1 or VCAM) are associated with leptospirosis-induced organ damage [43]. Chemokines along with endothelial adhesion molecules are induced by TNF-α, which promotes leukocyte attraction and extravasation into injured tissue.

Pentraxins are a class of evolutionarily conserved proteins that trace their evolutionary roots back to early invertebrates. C-reactive protein (CRP) and serum amyloid P component (SAP) are short pentraxins, and Pentraxin 3 (PTX-3) is a long one. In leptospirosis patients, increasing serum levels of PTX-3 were associated with mortality and disease severity [44].

The study of genetic susceptibility to infectious illness has experienced a revolutionary transformation in the previous decade [45]. Variations in the genetic makeup of the host may result in changes in susceptibility to leptospirosis [46]. Human leukocyte antigen (HLA), cytokine genes, and killer-cell immunoglobulin-like receptors (KIR) polymorphisms were studied in leptospirosis patients and healthy controls [41]. According to the researchers, susceptibility to leptospiral infection was significantly associated with alleles in the HLA-A and B loci and various HLA haplotypes. Patients with a history of leptospirosis exhibited considerably more polymorphisms in the IL-4 and IL-4R genes. Other researchers, on the other hand, discovered that leptospirosis infection was responsive to genetic diversity in the IL12RB1, IL1, and CISH genes [47]. TLR1 Ile602Ser and TLR2 Arg753Gln gene polymorphisms have also been found to substantially influence the development of severe leptospirosis with jaundice and hepatic insufficiency [48].

## 4. Immune Evasion in Leptospirosis

### 4.1. Neutrophils Are Poor Phagocytes

**Figure 2:**
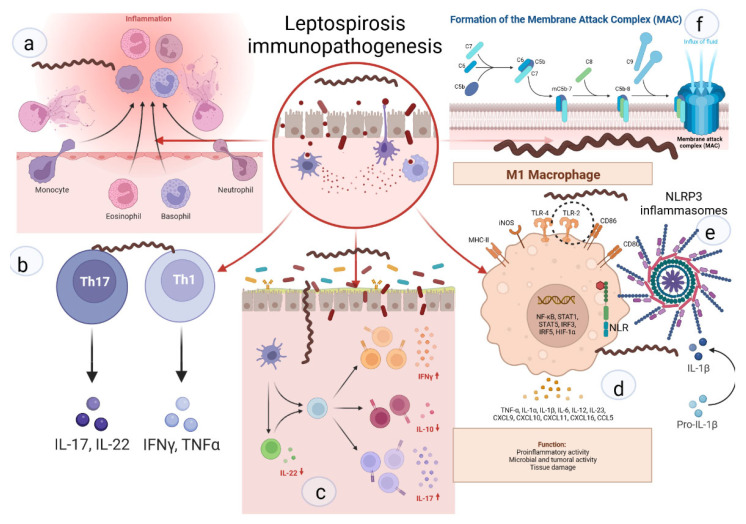
Key links in the immunopathogenesis of leptospirosis: (**a**) recruitment of neutrophils to the inflammatory zone and formation of NETs; (**b**) activation of differentiation of pro-inflammatory subpopulations of T-helpers; (**c**) increase in pro-inflammatory signaling against the background of deficiency of suppressor signals and Treg cells; (**d**) activation of M1-macrophages, stimulation of TLR2; (**e**) NLR with subsequent formation of NLRP3-inflammasome and subsequent maturation of IL1b; (**f**) -formation of MAC and destruction of the cell membrane.

Neutrophils are key cells that act against extracellular pathogens through three major mechanisms, i.e., phagocytosis, degranulation, and the release of extracellular traps (Figure 2). According to a recent study, most pathogenic leptospires were found on the neutrophil surface and were not phagocytized. Saprophytic leptospires, on the other hand, were taken up. Intracellular ROS levels were found to be related to leptospire uptake. Overall, pathogenic leptospires appear to be able to avoid or significantly reduce their uptake by human neutrophils, but the precise mechanisms and in vivo relevance involved are unknown [49].

Regarding degranulation, it has been shown that both virulent and avirulent *Leptospira* spp. are killed by both primary and secondary granule contents. The primary (azurophilic) granules, which contain myeloperoxidase (MPO), heparin-binding protein (HBP), defensins, and other antimicrobial peptides (AMPs), showed the highest microbicidal activity [50]. Remarkably, it has been recently shown that the *L. interrogans* outer membrane protein LipL21 is a potent inhibitor of neutrophil MPO [51]. This heme-containing peroxidase enzyme, which is primarily expressed in neutrophils, catalyzes the formation of ROS intermediates in the presence of hydrogen peroxide and halides such as hypochlorous acid (HOCl), a major effector of neutrophil-mediated microbial killing.

In regard to the NET formation, it was shown that *Leptospira* spp. could induce NET release in human neutrophils and that bacterial number, pathogenicity, and viability were important factors in NET release induction, whereas bacterial motility was not [52]. Interestingly, although NETs reduced leptospire survival, pathogenic but not saprophytic *Leptospira* spp. displayed nuclease activity and damaged DNA, suggesting that pathogenic leptospires may counteract this microbicidal mechanism [53].

### 4.2. Leptospiral Complement Evasion

Although leptospiral serum resistance to host complement was described many decades ago, the mechanisms underlying this resistance have only recently begun to be unraveled. Pathogens have developed complex strategies to avoid the immunological defense systems of a variety of hosts, including mechanisms to avoid complement activation and/or lytic complement attack. Among these mechanisms are the acquisition of host fluid-phase complement regulators, notably Factor H and C4b-binding protein (C4BP), the secretion of proteases that inactivate key complement components, and the expression of proteins in the pathogen surface that may inhibit or modulate complement activation [54]. The multifunctional LigB protein also binds to C3b and C4b and interferes with complement activation [55]. Lsa30, a novel leptospiral adhesion protein, may help pathogenic *Leptospira* to escape the immune system by interfering with the complement cascade through interaction with the C4bp regulator [56]. Lsa33 also binds to C4bp and may be important in immune evasion [57]. The recently described leptospiral complement regulator-acquiring protein A (LcpA) also binds to C4bp [58].

## 5. Immunological Aspects of Damage to the Kidneys, Liver, and Pulmonary Bleeding as the Basis of Weil’s Syndrome

### 5.1. Immunological Aspects of Renal Damage

The kidneys are the target organs in human leptospirosis pathology. Leptospirosis is associated with an overwhelming activation of inflammasomes and proinflammatory cytokines in the early phases, causing kidney inflammation and subsequent damage. On day 10 of the infection, Leptospira can be found in the proximal tubular cells, and in the tubular lumen on day 14 of the infection [59]. Its antigens are also found in the proximal tubular cells, macrophages, and the interstitium [60].

Leptospiral outer membrane proteins (OMPs) contain antigenic and virulent compounds, such as lipoproteins, LPS, and peptidoglycan that determine host responses [61]. Leptospiral LPS, found in OMPs, appears to be a major antigen affecting leptospiral immunity, and its function is thought to be related to host-pathogen interactions that determine virulence and pathogenesis. Leptospiral OMPs were extracted from cultured mouse renal epithelial cells, which displayed the expression of a variety of genes related to tubular cell injury and inflammation, to elucidate the mechanisms of tubule interstitial injury caused by Leptospira [62]. Nuclear transcription factor kappa B (NF-κB), activator protein-1, and several downstream genes expressed in the medullary thick ascending limb cells are all activated by the leptospiral OMPs [62]. LipL32, a key virulence lipoprotein on the OMP, induces tubulointerstitial nephritis-mediated gene expression in mouse proximal tubular cells and is a prominent immunogen during human leptospirosis infection [63]. Furthermore, LipL32, a hemolysin that causes erythrocyte hemolysis during leptospira infection, has a direct impact on the proximal tubular cells by significantly increasing the gene and protein expressions of some pro-inflammatory cytokines, such as inducible nitric oxide (iNOS), monocyte chemoattractant protein-1 (MCP-1), and TNF-α [64].

The effects of TLRs as the first line of immune defense mechanisms in the innate immune response were evaluated to determine whether TLRs could mediate the inflammatory response induced by leptospiral OMP in renal proximal tubular cells. Interestingly, only TLR2 but not TLR4 resulted in increased levels of iNOS and MCP-1 expression. As a result, the findings demonstrate that TLR2 is required for the early inflammatory response that occurs after Leptospiral OMPs, specifically LipL32, stimulate iNOS and MCP-1 in proximal tubular cells [65].

Leptospirosis induces the secretion of IL-1 and IL-18 by human macrophage cells through reactive oxygen species and cathepsin B-mediated activation of the NLRP3 inflammasome, which activates a cascade of inflammation in renal tubular cells [66]. Other circulatory cytokines and chemokines, including IL-6, IL-10, MCP-1, and TNF-α, are also produced during leptospirosis infection. An acute increase in cytokines and chemokines in patients with leptospirosis can lead to dangerous sepsis syndrome and severe sepsis due to an imbalance between pro- and anti-inflammatory reactions [67].

Thus, acute kidney injury (AKI) following leptospirosis can arise due to acute tubular necrosis (ATN) from ischemia and poor perfusion of renal tissue as the result of sepsis and septic shock. In addition, sepsis-associated AKI (sepsis-AKI) in leptospirosis is also possible, especially in patients with low blood pressure episodes. Pieces of evidence from both experimental and clinical studies show that septic shock develops into sepsis-induced immunosuppression, leading to the death of the host because of innate and adaptive immunity disturbances [68].

### 5.2. Pulmonary Involvement in Leptospirosis

The main cause of death in patients with leptospirosis is LPHS. The LPHS-related fatality rates are greater than 50% [69,70]. There are two main hypotheses for the pathogenesis of leptospirosis, a toxin-mediated mechanism, and host immune responses. The relatively low number of leptospires recovered from lung tissue suggests a possible role of circulating bacterial toxins produced at distant sites, such as the liver [71]. Although specific toxins are still unknown, a wide range of predicted proteases and hemolysins that may be involved in lung damage are encoded in the genomes of pathogenic Leptospira [72,73].

LPHS was established in a guinea pig model [74] using *Leptospira interrogans* serovar Copenhagen strains isolated from Brazilian patients who died from hemorrhage and acute respiratory failure [75]. This study showed that immunoglobulin and complement are deposited along the alveolar septa and that they can be associated with lung bleeding [74]. In addition, immunoglobulin deposits were observed in the alveolar septum and the alveolar space in a human case of LPHS [76].

In alveolar cells (pneumocytes), the active transport of Na to the interstitium by the α-Na-K-ATPase pump generates an osmotic driving force favorable to the entrance of Na from the alveolar lumen to pneumocyte through α-ENaC. The osmotic gradient between the lumen and the interstitial space promotes the movement of water via the paracellular pathway. Water also crosses the cell via a water channel (AQP5). Cellular volume is regulated primarily by electroneutral cotransporter NKCC1, which is found in virtually all cells and mediates coupled influx of Na, K, and chloride [77].

Some researchers showed that leptospirosis infection decreases α-ENaC protein expression in lung membranes of hamsters infected with leptospirosis. The investigators also found that basolateral protein expression of the Na-K-2Cl cotransporter NKCC1 was upregulated, as well as that AQP5 and α-Na-K-ATPase protein expressions were unchanged, in the lung tissue of hamsters infected with leptospirosis [78].

The decrease in ENaC and the increase in NKCC1 dissipate the osmotic gradient of Na between alveolar lumen and interstitium, leading to a decrease in water reabsorption in the intercellular space, leading to pulmonary edema. In human patients, leptospirosis has many presentations, including the severe pulmonary form (ARDS), which is characterized by impairment of the alveolar-capillary barrier. Impaired pulmonary fluid clearance resulting from downregulated α-ENaC expression, as well as the potential derangements related to increased NKCC1 expression, might have significant deleterious effects in the context of increased pulmonary permeability such as that observed in ARDS. Similar findings are observed in sepsis, a common cause of AKI and ALI [78].

### 5.3. Pathogenesis of Leptospirosis Liver Injury

It is known that the most severe form of leptospirosis, Weil disease, is also characterized by liver injury. Jaundice is an important manifestation of liver dysfunction, but its mechanism of leptospirosis has not yet been fully elucidated. The presence of jaundice implies a poor prognosis with a mortality rate of 19.1% [79].

Previous findings in patients recovering from leptospirosis, and thus out of the hemorrhagic phase of the disease, revealed non-specific hepatocellular damage affecting the sinusoidal pole, endoplasmic reticulum, mitochondria, and bile secretory apparatus [80,81]. The sinusoidal pole showed altered microvilli and the intercellular spaces were frequently widened with secondary microvilli and bile canaliculi dilated with partially or completely absent. The tight junctions were usually, but not always, preserved [82,83,84].

The pathogenic leptospires invade the intercellular junctions of host hepatocytes, and this invasion contributes to the disruption of the junction [85]. Bile leaks from the biliary capillaries to the blood circulation during the induction of jaundice. This is a novel mechanism of jaundice caused by leptospiral infection, based on previous electron microscopy studies in hamster experimental models of leptospiral infection [85].

The mechanism of jaundice proposed by Miyhara et al. is supported by the electron microscopy finding for leptospires and/or their remnants in the intercellular spaces of hepatocytes in humans, particularly in guinea pigs experimental disease [86].

### 5.4. Pathogenesis of Pancreatic Involvement in Leptospirosis

Acute pancreatitis was previously reported as an uncommon complication of leptospirosis [87]. The exact mechanism of acute pancreatitis in leptospirosis is not fully described. An immunological basis for pathogenesis of leptospirosis including TLR2 activation is described recently [88]. The most consistent pathologic finding in leptospirosis is a vasculitis of capillaries manifested by endothelial oedema, necrosis, and lymphocytic infiltration [89]. Small vessel vasculitis and ischemic injury leading to activation of proteolytic enzymes and auto-digestion is a possible mechanism [90].

### 5.5. Pathogenesis of Bleeding in Leptospirosis

Bleeding is a common symptom of severe leptospirosis. Although the cause of leptospirosis hemorrhages is not completely understood, some studies report ongoing fibrinolysis, activation of coagulation, impaired anticoagulation, and thrombocytopenia, whereas the involvement of disseminated intravascular coagulation is controversial [91,92,93].

**Figure 3:**
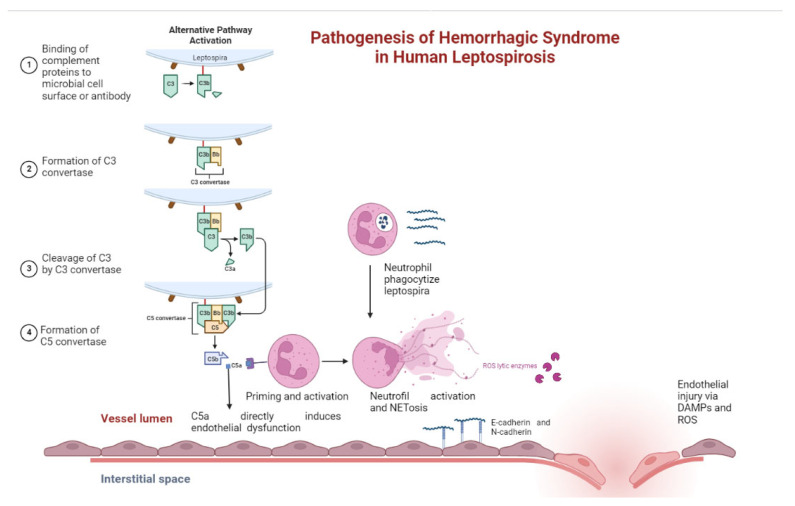
Pathogenesis of hemorrhagic syndrome in human leptospirosis.

Endothelial cell injury and vasculitis are generally accepted as major pathological characteristics of leptospirosis (Figure 3). Vasculitis is a condition characterized by inflammation of the vessel wall with reactive damage to mural structures causing endothelial cell injury. The clinical result may lead to intravascular thrombosis, subsequent organ infarction, and dysfunction.

Endothelial cells play a role in the pathophysiology of leptospirosis. The ability to attach to cadherins, a family of calcium-dependent transmembrane adhesion proteins that function to maintain cell–cell integrity and serve as receptors for *Leptospira interrogans*, is one of the mechanisms of adhesion to host cells [43]. Leptospira binds to vascular endothelial cadherin, which is found at intercellular junctions, as well as neural cadherin, which is found largely on the cell surface [94]. Both events can cause endothelial dysfunction, altered permeability, endothelial disruption, intercellular junction opening, and vascular leakage, all of which contribute to the development of hemorrhagic syndrome [80,95]. Multiple Leptospira adhesins have been reported to bind cells via cadherins or the extracellular matrix (ECM) molecules fibronectin, collagen, laminin, elastin, and plasminogen [96].

## 6. Gut Microbiota Involved in Leptospirosis

The human GI is an extremely diverse and complex microbial ecosystem with more than 10^14^ resident species interacting with the host and actively participating in many physiological processes, particularly in supporting the maintenance and development [97].

**Figure 4:**
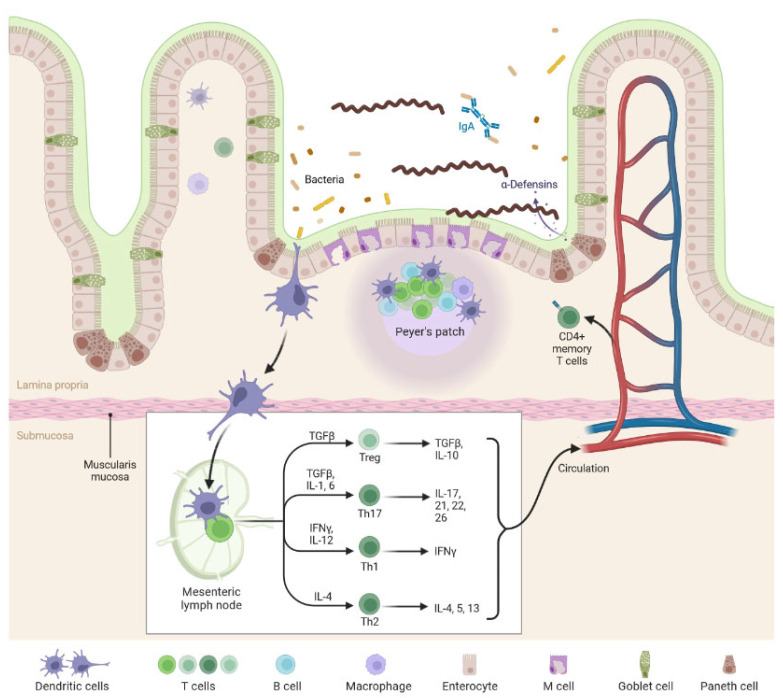
Commensal bacteria can influence the induction of intestinal T-helper cells in Gut-associated lymphoid tissue. Altered intestinal T helper cell profiles can have pathological consequences that are not limited to intestinal sites.

An imbalance in our gut microbiome is the cause of many diseases, including metabolic diseases, noncommunicable diseases, and infectious diseases [98,99]. It is well known that the gut microbiome plays a major role in initiating, adapting, and regulating the immune response [100,101]. The gut microbiota produces SCFAs that have anti-inflammatory properties, such as inducing apoptosis, inhibiting the tumor cell cycle, and preserving mucosal barriers to endotoxin infiltration [16]. Since most immune cells are located in the intestine, the gut microbiota plays a crucial role in the immune response of the intestine and other organs (Figure 4). Nowadays, there is growing evidence for the important link between the gut microbiome and other organs, such as the liver, the kidneys, and the lungs, which are most often damaged in leptospirosis [18,102,103]. The crosstalk between the gut and the lungs, kidneys, and liver is well-established, but the mechanisms by which the gut influences these organs or vice versa are unknown and relevant research is still in its early stages [104].

### 6.1. Gut–Kidney Axis

The gut microbiota produces many uremic solutes and toxins, such as indoxyl sulfate, and p-cresyl sulfate (PCS) during chronic kidney disease (CKD). However, increasing urea concentration results in a change in the gut microbiota. In patients with CKD, uremic toxins can cause renal anemia, pruritus, fatigue, mineral bone disorders, neurological damage, and cardiovascular impairment [105]. The pathogenic interconnection between the gut microbiota and kidney diseases is called the gut–kidney axis and appears to be involved in a wide range of clinical manifestations, such as CKD, acute kidney injury (AKI), hypertension, nephrolithiasis, hemodialysis, and peritoneal dialysis [106].

AKI is a prominent feature of leptospirosis, characterized by tubulointerstitial nephritis and tubular dysfunction. The non-oliguric hypokalemic form of AKI is an important sign of leptospiral nephropathy [107]. The incidence of AKI in patients with leptospirosis varies between 10% and 88%, depending on the definition of AKI, age, and severity of the disease [108].

AKI triggers immune responses, leading to epithelial disruption by activating macrophages and neutrophils of innate immunity and the T helper type 17 (Th17) cell from adaptive immunity [103]. These processes result in a leaky gut that causes dysbiosis. Studies have demonstrated that AKI causes gut dysbiosis in 24 h [109]. In contrast, gut dysbiosis also causes an imbalance of different chemicals, specifically uremic toxins, and SCFAs, ultimately altering immune and hormonal homeostasis and causing a worsened AKI [110].

### 6.2. Gut–Liver Axis

The gut-liver axis refers to the bidirectional relationship between the gut, along with its microbiota, and the liver, arising as a result of interactions between signals generated by dietary, genetic, and environmental factors [111]. The interconnection of the gut and the liver explains why disturbances in the intestinal barrier result in an increased portal influx of bacteria or their products to the liver, where they cause or influence a range of hepatic diseases [112].

Gut dysbiosis has been linked to liver diseases with distinct aetiologies, including acute liver injury, viral hepatitis, non-alcoholic fatty liver disease (NAFLD), alcohol-related liver disease, autoimmune hepatitis (AIH), primary biliary cholangitis (PBC), and primary sclerosing cholangitis (PSC) [113,114,115].

The liver is constantly exposed to bacterial components and microbial metabolites via the portal vein. LPS is a component of the cell wall of Gram-negative bacteria, and mild release of LPS from the gut can stimulate liver regeneration and tissue repair [116,117]. Furthermore, gut microbial metabolites, such as bile acids, SCFAs, and tryptophan metabolites, affect host metabolism and the immune system, which may indirectly influence liver injury and regeneration [118].

The role of gut-derived LPS as a cofactor in acute liver injury has been shown in models of acute liver injury induced by various hepatotoxic agents [119]. Under primary liver damage, gut-derived LPS can activate Kupffer cells to release pro-inflammatory mediators, such as TNF-α, interleukins (IL-1 and IL-10), lysosomal enzymes, and superoxide, which aggravate inflammatory responses and necrosis [115].

Gut-derived LPS is crucial for liver regeneration [116]. When the liver is subjected to an experimental physical or chemical injury, gut-derived LPS will pass through the compromised liver and spill into the general circulation, leading to low-grade systemic endotoxemia, which causes the production of hepatotropic factors, such as insulin, glucagon, epidermal growth factor (EGF), vasopressin and triiodothyronine (T3) [120].

SCFAs are important substrates for the integrity of the epithelial barrier, which limits the pro-inflammatory load on the liver. Butyrate enhances gut barrier function by increasing the expression levels of claudin-1 and zonula occludens-1 (ZO-1), decreasing LPS translocation and inhibiting downstream inflammatory responses [121]. Thus, SCFAs can indirectly affect liver damage and regeneration by maintaining the function of the intestinal barrier [122].

### 6.3. Gut–Lung Axis

The microbiota plays an essential role in the education, development, and function of the immune system, both locally and systemically [99]. Emerging experimental and epidemiological evidence highlights crucial cross-talk between the gut microbiota and the lungs, called the ‘gut–lung axis’ [123]. Changes in the constituents of the gut microbiota, through either diet, the disease is linked with altered immune responses and homeostasis in the airways [124]. The gut-lung axis is bidirectional, meaning that microbial metabolites and endotoxins from the gut can affect the lungs, while lung tissue inflammation can affect the gut microbiota [99].

An example of the influence of microbiota on the gut–lung axis is the relationship between intestinal SFB and pulmonary Th17 response. SFB can stimulate Th17 in the lungs and protect it from *Streptococcus pneumoniae* infection, enhancing lung mucosal immunity [125]. Dysbiosis of intestinal flora has been associated with respiratory diseases, such as asthma and cystic fibrosis [126,127]. These studies show that microbes play an essential role in cross-talk between the gut and the lungs and that microbial dysbiosis in the lungs may affect the homeostasis of the gut and vice versa [128].

### 6.4. Gut Microbiota and Leptospiral Infections

The role of the gut microbiota in leptospirosis infection was investigated using 16S rRNA sequencing, finding that the relative abundance of Firmicutes and Bacteroidetes between uninfected mice and leptospire-infected mice 7 days after infection differed significantly at the level of type and genus. Firmicutes/Bacteroidetes ratio (F/B Ratio) was significantly increased in the group of infected mice [13]. The study found that pre-depleting the gut microbiota of mice with antibiotics resulted in a significant increase in the burden of Leptospira in organs, such as the liver, kidneys, and lungs. Reverse effects were obtained with fecal microbiota transplantation (FMT) [13]. Changes in the gut microbiota may result from activation of intestinal immunity caused by *L. interrogans* infection. Host immune activation induced rapid transcriptional and metabolic adaptation of intestinal microbes [129].

**Figure 5:**
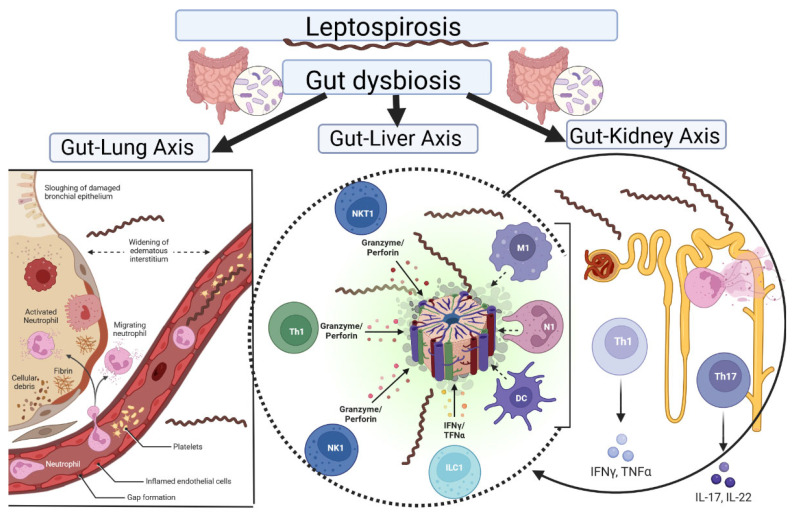
The influence of altered gut microbiota on the course of leptospirosis. According to our hypothesis, leptospirosis-induced gut dysbiosis can lead to a pro-inflammatory immune response, which can be one of the factors that lead to a severe course of leptospirosis.

Disturbance of the gut environment caused by infection results in disruption of the homeostasis between gut immunity and microbiota and eventually leads to an expansion or decrease of certain bacteria [130]. We proposed the hypothesis that gut dysbiosis caused by leptospirosis can lead to a more severe course of the disease, due to connections called axes (Figure 5).

## 7. Discussion and Conclusions

Leptospirosis is one of the most widespread and dangerous zoonoses in the world. The main cause of death in leptospirosis is the development of a severe form of leptospirosis—Weil’s disease, which affects the kidneys, lungs, and liver, which are a “triad” of target organs in leptospirosis.

Leptospires are capable of altering the gut microbiota. Similar mechanisms, according to which infectious agents change the gut microbiota, have also been revealed for SARS-CoV-2, Influenza, *M. tuberculosis*, and some other microorganisms [131,132,133,134]. Most of the current studies emphasize the possible influence of these microbiota changes on the course of diseases [135,136,137]. It was also found that changes in the gut microbiota are connected with various non-communicable diseases—for example, type 2 diabetes (T2D), cardiovascular disease, and obesity [138,139]. Data regarding T2D or arterial hypertension as an independent risk factor for severe leptospirosis remain controversial [140,141,142].

The gut microbiota can play a crucial role in directing immune responses outside the local environment, including the lungs, kidneys, and liver. This may be achieved by the systemic dissemination of metabolites, as has been shown for SCFAs [99]. These metabolites are produced in the colon but can reach other organs through the bloodstream, where they can exert their anti-inflammatory properties [143]. This complex interaction between the gut microbiota and the host’s immune system, which affects the body’s functions, has led to the formation of an “axis” between them [144]. This crosstalk occurs via an array of signalling pathways and direct chemical interactions between the host and the microbes, e.g., through microbial metabolites or individual bacterial species.

Therefore, the cross-connection of the gut microbiota with many internal organs of the body plays an important role in the clinical course of many diseases, including leptospirosis. Disturbance of the microbiota, such as antibiotic treatment, could increase susceptibility to *L. interrogans* infection. A whole line of research is being conducted to investigate the influence of gut microbiota on the course of leptospirosis and will expand the field soon.
